# Increased basolateral amygdala metabolic activity during flavor familiarization: an experimental study

**DOI:** 10.1186/s12993-023-00206-x

**Published:** 2023-02-03

**Authors:** Sergio Menchén-Márquez, María Banqueri, Beatriz Gómez-Chacón, Jorge L. Arias, Milagros Gallo

**Affiliations:** 1 Department of Psychobiology, Institute of Neurosciences, Center for Biomedical Research (CIBM), University of Granada, Parque Tecnológico de la Salud (PTS), Avda del Conocimiento, s/n, Armilla, 18016 Granada, Spain; 2 Instituto de Investigación Biosanitaria (IBS), Granada, Spain; 3 Laboratory of Neuroscience, Department of Psychology, University of Oviedo, Oviedo, Spain; 4 Centre for Discovery Brain Sciences, Edinburgh University, Edinburgh, UK; 5 Instituto de Neurociencias del Principado de Asturias (INEUROPA), University of Oviedo, Oviedo, Spain; 6 Department of Didactics, Area of Didactics of Experimental Sciences, Faculty of Education Sciences, University of Cádiz, Cádiz, Spain

**Keywords:** Amygdala, Attenuation of neophobia, Cytochrome c oxidase, Flavor, Neophobia, Taste

## Abstract

**Background:**

Novel flavors elicit a cautious neophobic response which is attenuated as the flavor becomes familiar and safe. The attenuation of neophobia reveals the formation of a safe memory. Previous lesion studies in rats have reported that basolateral amygdala integrity is required for taste neophobia, but not neophobia to flavor, i.e., taste linked to an odorous component. Accordingly, immunohistochemical analyses show that novel tastes induced higher basolateral amygdala activity when compared to familiar ones. However, a different role of basolateral amygdala in flavor attenuation of neophobia is suggested by lesion studies using a vinegar solution. Studies assessing basolateral amygdala activity during flavor attenuation of neophobia are lacking. Thus, we quantified cytochrome oxidase as an index of basolateral amygdala activity along the first and second vinegar exposures in order to assess flavor neophobia and attenuation of neophobia.

**Methods:**

We exposed adult male Wistar rats either once or twice to a 3% cider vinegar solution or water, and compared the basolateral amygdala, piriform cortex and caudate putamen brain metabolic activity using cytochrome c-oxidase histochemistry.

**Results:**

We found increased flavor intake and cytochrome c-oxidase histochemistry activity during the second exposure in basolateral amygdala, but not in the piriform cortex and caudate/putamen.

**Conclusions:**

The main finding of the study is that BLA metabolic activity was higher in the group exposed to a familiar vinegar solution than in the groups exposed to either water or a novel vinegar solution.

## Introduction

The ability to recognize familiar flavors which have been safe in previous encounters is critical for diet selection and survival. A novel flavor induces a neophobic response that is attenuated in a second exposure to the flavor without negative consequences, thus leading to increased consumption. The attenuation of neophobia (AN) requires the integrity of the perirhinal cortex (Prh), a brain area involved in taste and object recognition memory. Accordingly, Prh excitotoxic [1] and lidocaine-reversible lesions [2] interfere with the increased consumption during the second presentation of vinegar and saccharin solutions, respectively. We have also previously reported a similar impairment induced by basolateral amygdala (BLA) excitotoxic lesions. These lesions disrupt the pattern of Prh activity associated with AN during the second exposure to the vinegar solution [3]. Nonetheless, the critical mechanism disrupted is not yet known.

There is evidence supporting the BLA involvement in taste novelty detection. In fact, BLA permanent and reversible lesions have been found to interfere with neophobia to a 0.5% sodium saccharin solution [4, 5] and c-Fos expression induced in BLA by a novel saccharin solution is higher than that induced by a familiar one [6]. However, Lin and colleagues [4] did not find effects of BLA lesions on odor neophobia using a 0.1% amyl acetate solution. This is a relevant issue, since taste is rarely found isolated under natural conditions, but it is associated with other components, such as odor, becoming more appropriate to use the term “flavor”. Indeed, using a cider vinegar solution, a flavor which contains the acetic acid odorous component, we have reported increased activity during the second exposure in the nucleus accumbens shell [7] and the medial prefrontal cortex [8]. However, there is no data about the role of BLA in the vinegar familiarization process.

Cytochrome oxidase (CCO) is a marker of neural activity that indicates ATP increases and decreases according to the oxidative metabolic activity requirements. Thus, unlike other markers, CCO allows us to detect not only increases but decreases of brain activity. This technique has been previously applied to assess changes of the brain metabolic activity associated with taste familiarity on aversive conditioning using a latent inhibition procedure [9]. Likewise, CCO histochemistry has proven to be of great value to map functional brain networks involved in spatial learning acquisition [10, 11], retrieval [12] and extinction [11]. It is also sensitive to the effect of various treatments on spatial memory, such as social isolation [13], maternal separation [14], exercise [15] and pharmacological interventions [16].

Hence, CCO quantification during the first and second vinegar exposures would allow us to assess the potential BLA involvement either in the detection of flavor novelty or flavor familiarization. Increases of the BLA metabolic activity during the first flavor exposure would support a role in novelty detection. This would be consistent with the results found using a purely taste solution. On the other hand, increases of the BLA metabolic activity during the second flavor exposure would point to a role in flavor familiarization. This would be in accordance with the reported AN disruption by BLA lesions. Given the odorous component of the flavor used, the metabolic activity of the olfactory piriform cortex (Pir) as well as caudate/putamen (Cpu) as additional control areas was assessed. No sex differences have been reported either in vinegar neophobia or AN using the present behavioral procedure [17]. Hence, male rats were used for allowing comparison with previous results on brain activity, thus reducing the number of animals used. Additionally, the use of males avoids mildly invasive procedures required for assessing the estrus cycle.

## Material and methods

### Behavioral procedure

Twenty-one naive adult male Wistar rats were individually housed and maintained in a 12-h-light-dark cycle (8:00–20:00 h). Food was available ad libitum but access to water was restricted to the daily experimental 15-min drinking sessions at 10:00 h and to a daily additional 20-min rehydration session at 16:00 h. Water Baseline (BL) was recorded during the morning sessions in which the rats were handled for 3–5 min after BL1, BL3 and BL5 in order to avoid stress. After the water intake baseline, the animals were randomly assigned to one of the following groups: Novel (n = 8), Familiar (n = 5) and Control (n = 8). A cider vinegar solution (3%) was available during one (Novel) or two (Familiar) experimental sessions. The control group drank water throughout the experiment.

**Fig. 1:**
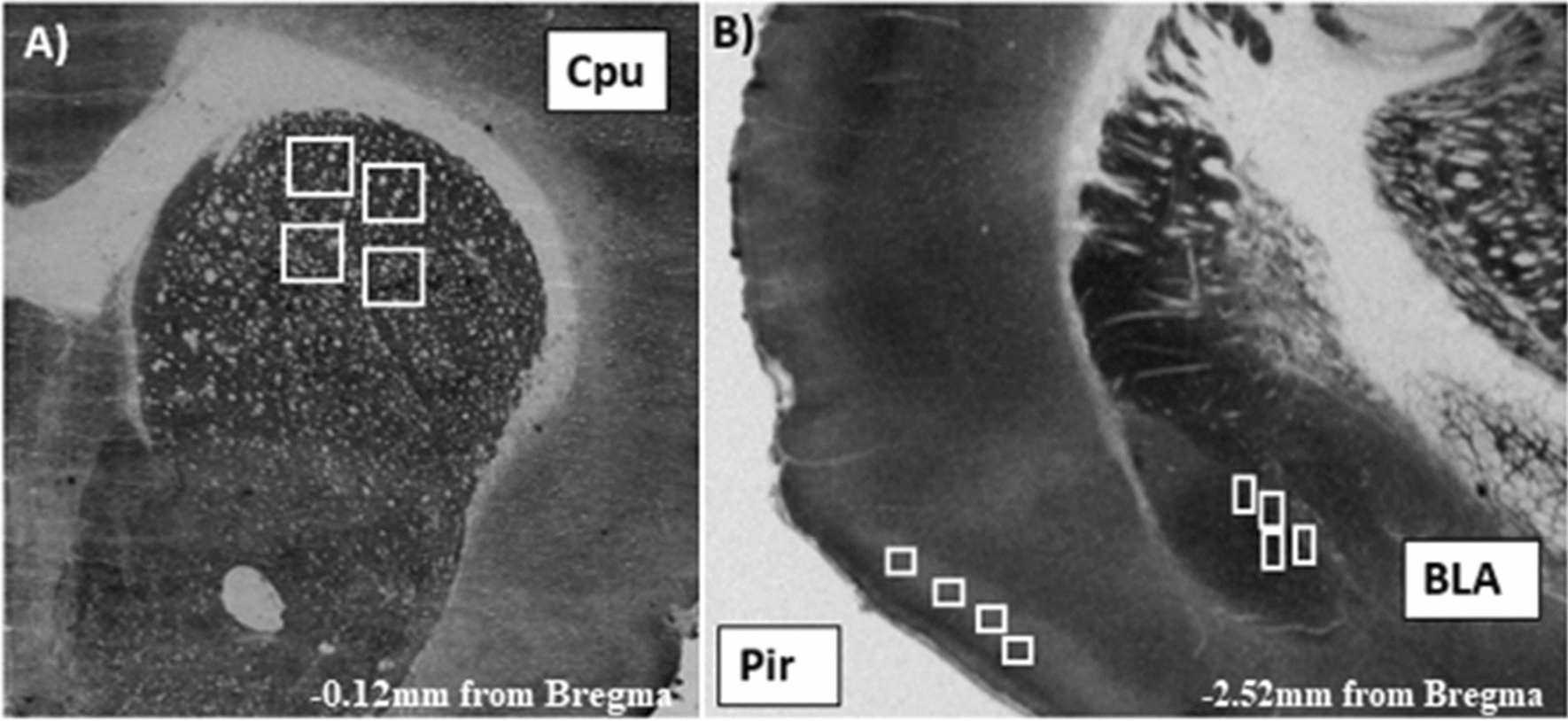
Anatomical location of Regions of Interest. BLA = Basolateral amygdala (− 2.52 mm), Cpu = Caudate/Putamen (− 0.12 mm), Pir = Piriform cortex (− 2.52 mm). Anteroposterior coordinates according to bregma [17]. All coordinates were taken from coronal brain slices

Ninety minutes after the experimental session each animal was sacrificed, the brain was removed and quickly frozen in isopentane (2-methylbutane; Sigma-Aldrich, Germany) to be stored at − 80 ºC. Brain coronal Sections (20 µm) were cut in a cryostat (Microm, HM505E, Germany). From each brain, sixty sections were taken in order to assess CCO in BLA, Cpu and Pir. The stereotaxic coordinates of the brain areas assessed according to the Paxinos and Watson atlas [18] are shown in Fig. 1.

The procedures were approved by the University of Granada Ethics Committee for Animal Research and by the Regional Ministry of Agriculture, Fisheries and Rural Development of Andalusia (17-02-15-195), following the ARRIVE guidelines and in accordance with the EU Directive 2010/63/EU for animal experiments.

### CCO histochemistry

The procedure for quantitative CCO histochemistry has been described elsewhere [13, 19]. In brief, after obtaining sets of tissue homogenate standards from the Wistar rat at different thicknesses (10, 30, 50 and 70 µm) in order to quantify the enzymatic activity, both sections and standards were incubated for 5 min in 0.1 M phosphate buffer (7.6 pH) with 10% sucrose (w/v) and 5% glutaraldehyde (v/v) (Merck, Germany). The slides were then rinsed 3 × in a 0.01 M phosphate buffer (7.6 pH) with 10% sucrose (w/v) and 0.05 M Tris buffer (7.6 pH) with 275 mg/L hexahydrated cobalt chloride, 10% sucrose (w/v), and 0.5% dimethylsulfoxide (v/v) for 10 min. This was followed by 1 × 0.01 M phosphate buffer (7.6 pH). Then, the sections and standards were incubated in 0.0075% cytochrome-c (w/v), 0.002% catalase (v/v), 5% sucrose (w/v), 0.25% dimethylsulfoxide (v/v) and 0.05% diaminobenzidine tetrahydrochloride (Sigma-Aldrich, Madrid, Spain). Both sections and standards were incubated for 5 min in 0.1 phosphate buffer (7.6 pH), at 37 ºC for 1 h. The reaction was stopped by fixing the tissue in buffered 4% (v/v) formalin 30 min RT. Finally, the slides were dehydrated, cleared with xylol and cover-slipped with Entellan (Merck, Germany). The intensity of the CCO staining was quantified through an optic densitometry analysis using a computerized image analysis system (MCID Elite, Interfocus Imaging Ltd., United Kingdom). The mean optical density (OD) of each region was measured in the right hemisphere using three consecutive sections. In each section, four non-overlapping readings were taken using a square-shaped sampling window that was adjusted for each region size, taking a total of 12 measurements per region and subject. These regions were averaged to obtain one mean per region for each animal. Then, OD values were converted to CCO activity units determined through the enzymatic activity of the standards measured spectrometrically. 

### Experimental design and statistical analyses

Flavor neophobia and AN were assessed using a two-factor mixed ANOVA design that includes a between-groups factor *Group* with 2 levels (*Control* group drinking water; *Familiar* group drinking vinegar twice) and a within-subjects factor *Days* with 3 levels (Water baseline, first vinegar exposure, second vinegar exposure), being the rat ID the random factor (animals were randomly assigned to groups). Post-hoc Bonferroni tests were applied.

Given the need to sacrifice the animals for assessing brain CCO activity, an additional Novel group was required. Thus, a two-factor mixed ANOVA design was applied including a between-groups factor *Group* with 3 levels (Control group drinking water; Novel group drinking vinegar once, Familiar group drinking vinegar twice) and a within-subjects factor *Region of Interest (ROI)* with 3 levels (BLA, Cpu, Pir). Again, since animals were randomly assigned to the groups, rat ID was the random factor. Post-hoc comparisons were performed with post-hoc Bonferroni tests.

## Results

**Fig. 2:**
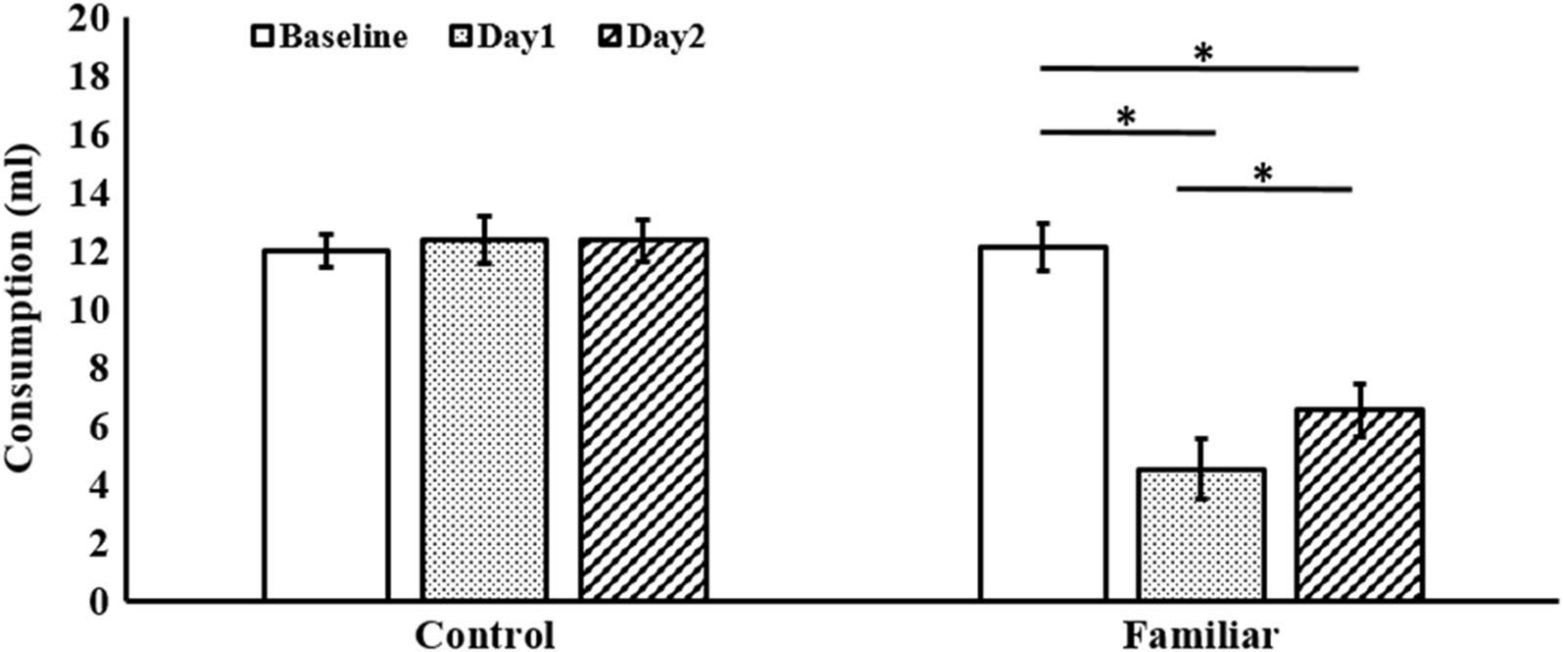
Mean (± SEM) consumption of each group during the water baseline, the first and the second exposure to water (Control) or cider vinegar solution (Familiar). * = *p* < 0.05

Figure 2 shows the mean (± SEM) intake of the Familiar group drinking the 3% cider vinegar solution that evidence the neophobic response and its attenuation on the second presentation in comparison with the Control group drinking water. A mixed 2 (Group) × 3 (Days) ANOVA analysis showed a within-subject Day effect [*F*(2, 18) = 41.121, *p* = 0.001, *η*_*p*_^*2*^ = 0.820] and interaction of Group × Day [*F*(2, 18) = 61.407, *p* < 0.001,* η*_*p*_^*2*^ = 0.872]. A Group effect was found too [*F*(1, 9) = 23.982, *p* < 0.002, *η*_*p*_^*2*^ = 0.727]. Levene’s test showed homogeneity of variances for all within-subject variables. Post-hoc Bonferroni tests indicated that Group interaction is due to the Familiar group drank a lower amount of the novel vinegar solution on Day1 than both the water (Baseline) (*p* < 0.001) and the familiar vinegar solution on Day2 (*p* < 0.03). No differences were found in the Control group (*p* > 0.05).

**Fig. 3:**
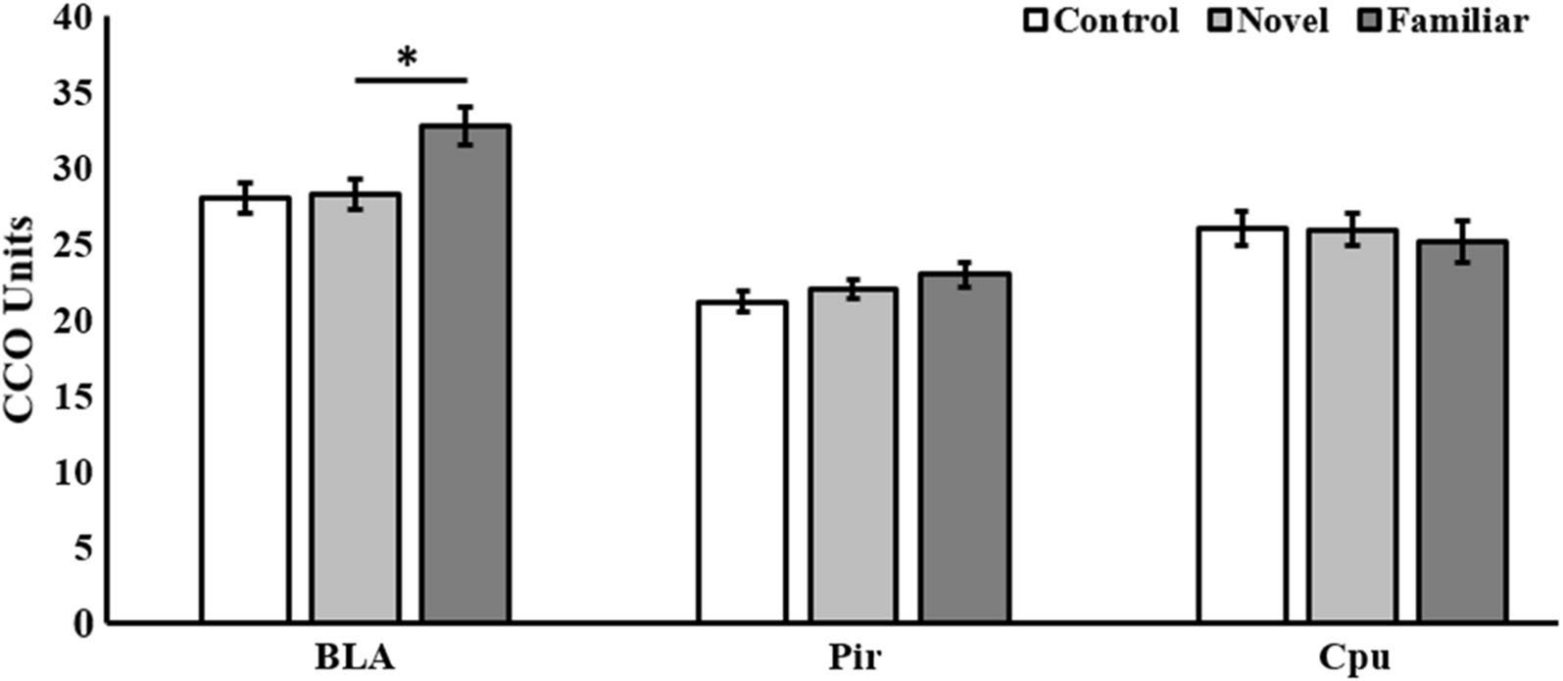
Mean (± SEM) brain metabolic activity of each region of interest (BLA, Pir, Cpu) when exposed to water (Control) or to a cider vinegar solution either once (Novel) or twice (Familiar). * = *p* < 0.05

Regarding the CCO results, Fig. 3 show the mean (± SEM) CCO units in each region of interest of the three groups (*Control, Novel and Familiar*). A mixed 3 (Group) × 3 (ROI) ANOVA analysis was carried out. Levene’s tests showed homogeneity of variances for all within-subject variables. The analysis revealed a significant effect of ROI, [*F*(2,36) = 38.858, *p* < 0.001, *η*_*p*_^*2*^ = 0.179]. Neither the interaction Group × ROI [*F*(4, 36) = 1.964, *p* > 0.05, *η*_*p*_^*2*^ = 0.683] nor the main factor Group [*F*(2, 18) = 3.235, *p* > 0.05, *η*_*p*_^*2*^  = 0.264] were significant. Post-hoc Bonferroni tests showed higher metabolic activity in BLA than Pir (*p* < 0.001) and Cpu (*p* < 0.01), being Pir activity higher than Cpu (*p* < 0.005). Although the interaction was not significant, one-way ANOVA analyses were applied to each ROI because testing our hypotheses required gaining knowledge about the difference between groups in BLA. They indicated significant differences only in BLA [*F*(2,18) = 5.365, *p* < 0.05, *η*_*p*_^*2*^ = 0.373], but not in Pir [*F*(2,18) = 1.374, *p* > 0.05, *η*_*p*_^*2*^ = 0.132] or Cpu [*F*(2,18) = 0.148, *p* > 0.05, *η*_*p*_^*2*^ = 0.016]. Post-hoc Bonferroni tests showed that the Familiar group exhibited higher metabolic BLA activity than both Novel group drinking vinegar for the first time (*p* < 0.05) and Control group drinking water (*p* < 0.05). There were no differences between Novel and Control groups (*p* > 0.05).

## Discussion

The behavioral results are consistent with those previously reported [3]. Flavor neophobia and AN are evidenced by reduced intake of the novel vinegar solution which significantly increases on the second exposure as the flavor becomes familiar. The results indicate higher BLA metabolic activity in the group exposed twice to the vinegar solution than that exposed once. This supports a selective involvement of the area in the processes leading to the formation of the safe taste memory but not novelty detection. Our results do not allow us to draw conclusions regarding the specific process associated with BLA activity. A potential role of BLA in the retrieval of the flavor memory formed during the first exposure is conceivable. Both retrieval and stabilization of the safe memory should be taking place in the second flavor exposure. Hence, it is feasible an association between the reported increase in BLA activity and memory retrieval. In fact, memory formation should have been initiated during the first exposure but BLA activity changes were not found at this stage. Also, the increased BLA activity during the second vinegar exposure could be associated with a selective stabilization process.

Moreover, since no significant effects were found in Pir, this undercuts the hypothesis that the increased energy expenditure in BLA could be due to enhanced olfactory processing during the second exposure. This is consistent with our previous findings using c-Fos immunohistochemistry [20]. We did not find significant differences between the activity induced by one and two vinegar exposures in neither the anterior nor the posterior piriform cortex regions evaluated. In fact, we have previously reported increased activity induced by drinking a well familiarized vinegar solution after six exposures, but not after two, only in the rostral part of the posterior Pir [20]. This region corresponds to the level assessed in the present study. Thus, the absence of differences after two exposures confirms no role of Pir during the familiarization process. Furthermore, the fact that no effect of flavor exposures was found in Cpu corroborates a selective BLA involvement in flavor familiarization.

These findings suggest that neophobia and AN might be independent processes that rely on dissociable brain areas. Accordingly, there was a selective increase of BLA activity in those animals drinking the familiar vinegar solution in comparison with those drinking water while there were no changes in those drinking the novel vinegar solution. We have previously reported similar results in the nucleus accumbens shell [7] and the medial prefrontal cortex [8]. Although our results do not allow us to draw a circuit approach, it is conceivable that BLA would form a functional network with these areas. Hence, we cannot discard that the reported BLA activity changes during AN could be driven by top-down control from areas such as the prefrontal cortex. Previous reports indicated higher BLA activity during drinking a novel saccharin solution [6] but no effect of BLA lesions on odor neophobia using a 0.1% amyl acetate solution [4]. The present findings would suggest the involvement of BLA in flavor familiarization but not novelty detection. Thus, those flavors composed of an odorous component would involve BLA activity during the formation of the flavor safe memory.

Our results confirm the value of the CCO technique to explore changes associated with learning and memory. Hence, flavor recognition memory is added to taste and spatial learning in which CCO has proven to be a useful technique for identifying the brain areas involved. In fact, previous reports indicated increased CCO activity in BLA during the first day of training in a spatial learning task [21].

Taken together, our results would support a relevant role of the amygdala either in retrieval and/or the stabilization process leading to a safe flavor memory.

## Conclusions

The main finding of the study is that BLA metabolic activity was higher in the group exposed to a familiar vinegar solution than in the groups exposed to either water or a novel vinegar solution. These results are consistent with results of lesion studies and support the basolateral amygdala involvement in those processes leading to the attenuation of flavor neophobia.

## Data Availability

The data that support the findings of this study are available from the corresponding author upon reasonable request.

## Abbreviations

AN: Attenuation of neophobia
BLA: Basolateral amygdala
CCO: Cytochrome oxidase
Cpu: Caudate/putamen
OD: Optical density
Pir: Piriform cortex
Prh: Perirhinal cortex
ROI: Region of interest
